# The origins and function of musical performance

**DOI:** 10.3389/fpsyg.2023.1257390

**Published:** 2023-11-10

**Authors:** Robin I. M. Dunbar

**Affiliations:** Department of Experimental Psychology, University of Oxford, Radcliffe Quarter, Oxford, United Kingdom

**Keywords:** singing, dancing, endorphins, social bonding, archaic humans

## Abstract

Music is widely recognised as a human universal, yet there is no agreed explanation for its function, or why and when it evolved. I summarise experimental evidence that the primary function of musicking lies in social bonding, both at the dyadic and community levels, via the effect that performing any form of music has on the brain’s endorphin system (the principal neurohormonal basis for social bonding in primates). The many other functions associated with music-making (mate choice, pleasure, coalition signalling, etc) are all better understood as derivative of this, either as secondary selection pressures or as windows of evolutionary opportunity (exaptations). If music’s function is primarily as an adjunct of the social bonding mechanism (a feature it shares with laughter, feasting, storytelling and the rituals of religion), then reverse engineering the problem suggests that the capacity for music-making most likely evolved with the appearance of archaic humans. This agrees well with anatomical evidence for the capacity to sing.

## Introduction

The capacity to make and enjoy music is a human universal: there is no society or culture that does not have some form of music-making. Inevitably this raises questions about the function of music and its evolutionary origins. The very richness of music-making across human societies has, however, resulted in the discussion becoming mired in a plethora of alternative proposals. These have included the role of music-making in mate advertising (Darwin, 1871; Miller, 2000; Kaskatis, 2006), coordinating emotional states in groups of people (Roederer, 1984), coordinating (male) war bands (Hagen and Bryant, 2003), coalition signalling (Mehr et al., 2021) and social bonding (Dunbar, 2012; Dunbar et al., 2012; Savage et al., 2015, 2021). When many alternative explanations are offered for a trait, all of which can claim some empirical evidence in their support, it is often because they all capture some aspect of reality. Some reflect genuine functional explanations, others reflect secondary benefits or windows of evolutionary opportunity (such as sexual selection acting on a trait that evolved for another purpose), while yet others may offer mechanisms explanations. Disentangling these can often be challenging, but failure to do so invariably causes profound confusion (Dunbar and Shultz, 2023a).

A second issue that has bedevilled discussions of this topic has been a naïve understanding of biological fitness (and the processes that underpin selection; Dunbar and Shultz, 2023a). There has been a longstanding tendency to view fitness only in terms of direct benefits to the individual: a longer tail makes a male peacock more competitive in obtaining matings with females. This, at root, was the essence of Darwin’s theory of evolution by natural selection. What Darwin did not fully appreciate was the role of sociality in advanced mammals and birds. Group-living introduces a new layer of processes that influence an individual’s fitness – reflected a century after Darwin in Hamilton’s (1964) conception of inclusive fitness (or more correctly *neighbour modulated fitness*). The skills needed to create a stable group so as to gain the benefits that derive from living in a group become a crucial part of the mix of strategies that give rise to an individuals’ personal (inclusive) fitness.

In part, this is derivative of a failure to fully appreciate the nature of sociality in highly social species. There is a widespread assumption (especially among those with limited knowledge of natural history) that group-living is a trivial by-product of the environment in which individual species happen to find themselves. However, there is an important, and longstanding, distinction in behavioural ecology between aggregations (herds, flocks) and congregations (stable social groups). Creating stable groups is extremely challenging for animals (and humans) because, all else equal, the stresses of living in close proximity with others create strong dispersive forces that otherwise limit social group size (Dunbar and Shultz, 2021a). To counteract these forces so as to be able to live in large groups, anthropoid primates evolved grooming-based alliances that help buffer individuals against these pressures. This creates a multi-layered structure to (inclusive) fitness that needs to be carefully teased apart in order to avoid confounding levels of explanation.

Here I focus on the principal role that music plays in the human context, in particular its function as a mechanism for creating large bonded social groups. Whether it evolved *de novo* for this purpose or this function was exapted from some prior function is an interesting question that I shall consider only very briefly. To set the scene, I first summarise the evolutionary context in which music evolved. I then provide evidence that music subserves a social bonding function by up-regulating the brain’s endorphin system. Finally, I use a reverse engineering approach to determine when this function most likely evolved.

## The context of primate sociality

The decision to live in stable, bonded social groups was not merely key to primate (and human) evolution but was by far the most difficult evolutionary transition to achieve. It remains the most challenging thing that primates have to do on a daily basis. The central problem for all mammals is that, all else equal, groups will inevitably disperse as time passes. This is a consequence of two distinct forces. One is the intense stresses that living in close proximity to others engenders (Dunbar and Shultz, 2021a); the other is the fact that, all else equal, groups will naturally drift apart as individuals’ activity cycles get out of synchrony and one wants to rest while another wants to continue feeding. We see the second most clearly in herding species, where groups convene and disperse on an hourly basis. Despite being the most intensely social of all the primates, baboon groups are more likely to disperse and break up when day journeys are long and group size is large (King and Cowlishaw, 2009; Dunbar, 2023; Dunbar and Shultz, 2023b). These stress effects are much less obvious to the naïve observer, mainly because most social species have evolved behavioural strategies to counteract their effects. We usually see their effects only on the rare occasions when a population lives in unusually large groups. However, more fine-grained analysis does demonstrate that females suffer significant fertility effects as group size increases (Dunbar and Shultz, 2021a).

Anthropoid primates (and therefore humans) counteract these dispersive effects by forming bonded relationships, based on social grooming. This creates what is in effect a gravitational field that holds individuals in place near their grooming partners (Dunbar, 2023). Primate relationships are formed through a dual process mechanism – two separate cognitive processes that exploit different neural pathways which work together in tandem to build an emotionally intense relationship (in effect, a friendship; Dunbar, 2018). One pathway involves the brain’s endorphin system and is activated by social grooming. Endorphins are part of the brain’s pain management system (Zubieta et al., 2001). They are opioids, and hence chemically closely related to morphine and other opiates (but without the destructively addictive properties). They create a sense of calmness and relaxation that gives rise to feelings of warmth and trust towards those with whom one grooms (Machin and Dunbar, 2011). The endorphin system is activated by a highly specialised peripheral neural system, the afferent c-tactile neurons that respond to one stimulus and one stimulus only – light slow stroking at 3 cm per sec (about the speed of hand movements during grooming; Löken et al., 2009). The receptors for these nerves are distributed throughout the hairy skin (Vallbo et al., 1993; Olausson et al., 2010) and are activated by the deflection of the skin that occurs when hands move across it parting the fur during grooming. In effect, grooming creates an emotionally intense relationship that compels an individual both to keep close to its partner and to come to that partner’s aid when it is under attack from another animal (Dunbar, 1980).

The endorphin system essentially sets up a psychopharmacological platform off which the second component of this dual process mechanism functions, namely the cognitive capacity to build and manage relationships (as reflected in the social brain hypothesis: Dunbar, 1998; Shultz and Dunbar, 2022). This involves two key skills that are specific to the anthropoid primates, namely the ability to mentalise (the capacity to understand the intentions of others so as to predict their behaviour more accurately, including the capacity to manage third party relationships) and the ability to inhibit prepotent actions (i.e., self-control; Dunbar and Shultz, 2021b, 2023b; Dunbar, 2023). These allow individuals to decide when another’s actions are accidental or malicious, and when to press an advantage or hold back (either because pursuing an action would, in effect, destabilise relationships or because it would cause an opponent’s allies to come to its aid). This is not to suggest that primates think through these issues consciously, any more than humans do in the heat of the moment; but these are, in effect, the decision processes that underpin the moment-by-moment unfolding of a social interaction. Getting it wrong means destabilising the fine balance on which group stability hangs, and thus risks driving other individuals out of the group, resulting in a negative downward pressure on group size. These abilities are cognitively very demanding (Lewis et al., 2017; Dunbar and Shultz, 2021b). In primates, managing relationships involves a very large connectome (the default mode neural network) that integrates processing in a number of large, distributed units in the prefrontal cortex, the temporo-parietal junction and the temporal lobe, with connections down into the limbic system and the cerebellum (Mars et al., 2012; Horn et al., 2014; Xu et al., 2020; Sandhu et al., 2021; Smallwood et al., 2021). The default mode network makes up a very large proportion of the primate brain (and the neocortex, in particular), and largely explains the social brain relationship.

The problem with grooming, as the principal primate bonding mechanism, is that it is very expensive in terms of time. In intensely social species like gelada baboons, it amounts to something like 25 min per day *per bonded dyad* (a quantity very similar to that invested in their closest relationships by humans: Sutcliffe et al., 2012). Moreover, its physical intimacy limits the number of individuals that can be groomed at any one time to just one, with complex multi-party grooming groups being rare (occasional claims to the contrary notwithstanding) in monkeys, apes and even humans (Dunbar, 2022a). Given that primates do not have unlimited time available for grooming (Dunbar et al., 2009), this effectively limits the size of social group that can be bonded together by grooming to about 50 individuals (Dunbar, 2022a). No primate species has a mean group size larger than this (Dunbar et al., 2018).

Hominins faced an inevitable dilemma once they began to increase group size above this limit up towards the size that now typifies modern humans (ca. 150: Dunbar, 2020). They could not increase grooming time to accommodate larger numbers of individuals since australopithecine time budgets were already at their absolute limit (Bettridge, 2010; Bettridge and Dunbar, 2023, submitted). The solution seems to have been to find other behaviours that activated the endorphin system in the same way social grooming does without needing to involve direct physical contact. Over a period of several million years, hominins added a number of behaviours that now constitute our social toolkit: these include laughter, singing (in the sense of chorusing without words), dancing, feasting, storytelling and the rituals of religion (Dunbar, 2014, 2022b). All of these have been shown to activate the endorphin system, and all of them have been shown to enhance the sense of bonding (Dunbar, 2021a). They are likely to have been adopted piecemeal rather than all at once, not least because there is good evidence to suggest that laughter long predates the others, while feasting, storytelling and religion (which all depend on language) must necessarily postdate the evolution of fully modern language (and this is likely to have been late: Dunbar, 2009).

Here I focus on the role that music might have played in this sequence. I first establish that the various forms of musicking (singing, dancing, performing on instruments) do activate the endorphin system and enhance feelings of bondedness. For this, I summarise empirical evidence from a large number of experimental studies. I then ask whether we can say anything at all about the timing of the origin of musicking. To do this, I set its function (community bonding) into a framework based on the time demands of social bonding. For this, I use a reverse engineering approach: we seek, first, to establish the all-else-equal demands of the bonding mechanism in the absence of any subsequent adaptations, then ask (1) how musicking might have filled the gaps that this identifies and (2) when this step became essential if humans were to be able to continue increasing the size of their groups. In effect, we ask not what benefit music-making might have had, but what the consequences would have been at each successive time point if the capacity for music-making had not evolved at that particular point in time.

## Does music facilitate social bonding?

Over the past decade, we have undertaken an extensive series of experiments on the effects of the six social behaviours on the brain’s reward system in relation to their role in community bonding. Our focus has been on endorphins and, to a much lesser extent, dopamine (which is biologically coactive with the endorphin system). We focus on endorphins because of the psychopharmacological evidence identifying this neuroendocrine as fundamental to primate social bonding (e.g., Keverne et al., 1989). Although much has been made of oxytocin (and occasionally serotonin and vasopressin) in this context, in fact the evidence is at best unconvincing (other than for a role in reproductive pairbonds), whereas endorphins and dopamine combine to influence both social predisposition and wider network aspects of relationships, as well as playing a significant role in pairbond dynamics (Pearce et al., 2017, 2019). More importantly, endorphins are naturally better designed to support longlasting relationships: the endorphin half-life is measured in hours (Rossier et al., 1977; Aronin et al., 1981), whereas that of oxytocin is measured in minutes (Homeida and Cooke, 1984; Wachs et al., 1984; Paccamonti et al., 1999).

**Figure 1:**
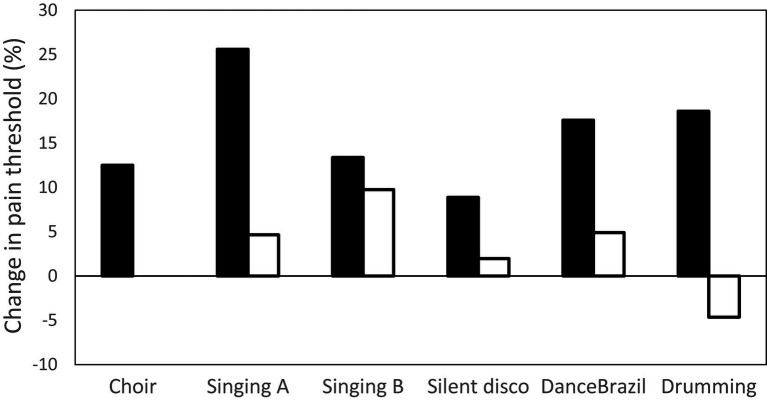
Six experiments testing endorphin up-regulation (indexed by change in pain threshold from before to after activity session) due to musical activity. Bars indicate mean change in pain threshold for an experiment. Filled bars: experimental groups; unfilled bars: control groups. Choir: singing practice in an amateur choir of 20 (Weinstein et al., 2016); Singing A: choir practice and church service; control condition: prayer meeting (Dunbar et al., 2012); Singing B: amateur singing classes; control condition: hobby classes (Pearce et al., 2015); Silent disco: dance experiment, groups of four with full body movements in synchrony; control condition: arm movements only, not in synchrony (Tarr et al., 2016); DanceBrazil: dance experiment in Brazil, groups of three, full body movements in synchrony; control condition: arm movements only, not in synchrony (Tarr et al., 2015); Drumming: drumming circle practice session; control condition: listening to music while working (Dunbar et al., 2012). Pain thresholds determined by a cold pressor task.

Figure 1 summarises the results from the experiments that tested the hypothesis that different kinds of musical performance up-regulate endorphins. In each case, endorphin up-regulation is indexed by the percentage change in pain threshold either side of an activity (a commonly used proxy for endorphin activation; filled bars). The tasks represent a range of musical activities, including singing, dancing, and performing on instruments (drumming), all of which were performed in groups ranging in size from 3–20 individuals. Except for the choir study, each experimental task is compared to a non-musical control group (the activities are listed in the figure legend; unfilled bars). It is obvious that musical activity, whether singing, dancing or performing, elevates pain thresholds, whereas control activities do so at a much reduced level or may even have a negative effect (a common finding for many non-musical activities: Dunbar et al., 2012, 2016a,b).

**Figure 2:**
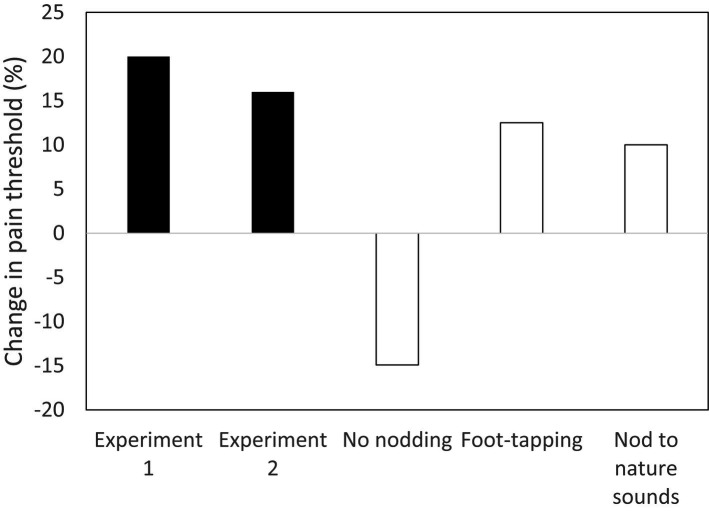
Two experiments testing whether head-nodding in time to music up-regulates brain endorphin system (indexed by change in pain threshold from before to after activity). Experimental conditions: Experiment 1 and Experiment 2 (nodding head in time to music while listening to a compilation of popular music). Control conditions: No nodding (listening to same music sequence sitting completely still); Foot-tapping (listening to same music sequence, while tapping one foot in time to music); Nature sounds (nodding head while listening to compilation of arhythmic nature sounds). Source: Dunbar et al. (2021a,b).

Figure 2 summarises two experiments that tested whether head-nodding in time to music (a common behaviour among those listening to music) was sufficient to activate the endorphin system. It was motivated by a recent finding that the mammalian cochlea has an unusually high density of the same kind of unmyelinated type-II spiral-ganglion (SGN) receptors that underpin the c-tactile neurons in the skin (Zhang and Coate, 2017; Wu et al., 2018). Participants were asked to nod their head (and no other part of the body) in time to a 13-min seamless compilation of popular music heard through earphones. The three control groups were asked either to remain completely still while listening to the same music, to tap a foot in time to the music or to nod the head to a compilation of (arhythmic) nature sounds (wind, falling water, bird song). In this case, pain thresholds were measured using the wall-sit (or Roman chair) task, which is broadly considered a more reliable assay of pain tolerance because the participant has to hold a painful position (a standard skiing exercise) until they collapse onto the floor. Compared to the main control condition (no nodding), pain thresholds increased significantly following head nodding when listening to music. Notice that both foot-tapping to the music and nodding the head while listening to nature sounds generated an uplift in pain threshold, but that in neither case was this uplift as large as that in the experimental condition (when nodding was rhythmic). It appears to be this same effect that underpins the calming effect that rocking and stroking has on distressed babies (Gursul et al., 2018; Yadav et al., 2022; Manzotti et al., 2023).

That the change in pain threshold actually does reflect endorphin up-regulation has been confirmed using positron emission tomography (PET) to measure μ-receptor endorphin uptake in response to both grooming (Nummenmaa et al., 2016) and laughter (Manninen et al., 2017). In one set of dance studies, we ran a control condition in which half the participants were selected at random to receive an endorphin-blocker (naltrexone) before undertaking the musical exercise. Naltrexone locks onto the endorphin receptors, but is pharmacologically neutral. Since this prevents uptake of endorphins when these are released during the subsequent activity, the effect on pain thresholds is the same as if there was no endorphin activation. The result was a significant reduction in pain threshold comparable to that seen in control groups engaged in a low intensity, unsynchronised, arms-only dance activity (Tarr et al., 2017). That communal singing might activate the endorphin system has also been confirmed in birds. The song-like chorusing (“flock talk”) that occurs in many bird species that form cohesive, stable social groups (e.g., Guinea fowl, starlings, babblers) has also recently been shown to elevate endorphin levels (as indexed by change in pain thresholds; Riters et al., 2019).

**Figure 3:**
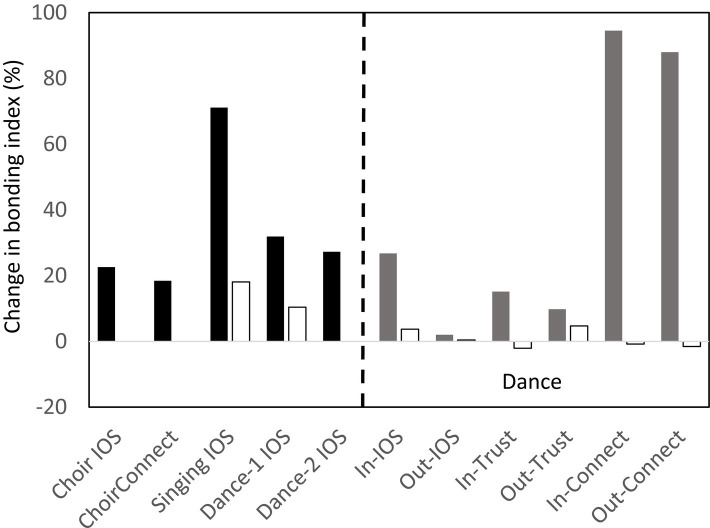
Mean change in bonding index before to after a musical activity, referenced to other members of the experiment (IOS or In-IOS) or the participant’s wider social circle not present at experiment (Connect or Out-IOS). Bonding index is either Aron et al. (1992) IOS scale or a self-rated analogue 1–10 scale of perceived bondedness to target group. Lefthand side (black bars): ChoirIOS and ChoirConnect: singing practice in an amateur choir of 20 (Weinstein et al., 2016); Singing IOS: amateur singing classes, with hobby classes as control group (Pearce et al., 2015); Dance-1: amateur dancing classes, with control group as gym classes (Tarr and Dunbar, 2023); Dance-2: amateur dance classes (Dunbar et al., 2012). Righthand side (grey bars): amateur dance classes, with gym sessions as control (Tarr and Dunbar, 2023). Trust in others (1–10 analogue scale); Connect: feelings of connectedness to others (1–10 analogue scale). IN: index relative to other members of class; OUT: index relative to participant’s wider social circle not present at experiment.

In some of our studies, participants were asked to rate their emotional closeness to other members of the group before and after the musical task. Bondedness was indexed by the Aron et al. (1992) IOS (Inclusion-of-Other-in-Self Scale), a 7-point Likert-type scale of how emotionally close the participant feels to other members of their group, referenced either to the experimental group or to the wider community from which that group was drawn. In two of the studies, participants also rated how connected they felt to the same reference groups and how much they trusted the other members of the group, with both indexed on a 1–10 analogue scale. The results are given in Figure 3. Again, the contrast is between a musical activity (filled bars) and a control group (a non-musical activity or an unsynchronised, low-effort musical performance; unfilled bars). As with the pain thresholds, it is obvious that bondedness ratings are consistently (and usually substantially) higher after taking part in a musical activity than after a more neutral activity. It is notable that the increase in the IOS bondedness index, connectivity and trust were higher in respect of those with whom the participant had just danced (in this particular study) than to other members of their immediate community not actually present for the experiment. In other words, the effect of the endorphin activation seems to be very specific to the individuals actually present, whether these are strangers or friends. In effect, musicking together can turn strangers into instant friends (the “Icebreaker Effect”: Pearce et al., 2015), even though it has no impact on the quality of the relationships with friends when these are not actually present. Further evidence that music has a strong social component is given by Nummenmaa et al. (2016), who found that the brain regions activated while listening to music strongly overlap those involved in more explicitly social behaviours such as stroking or laughter, as well as with the regions that have a strong endorphin response to these behaviours.

**Figure 4:**
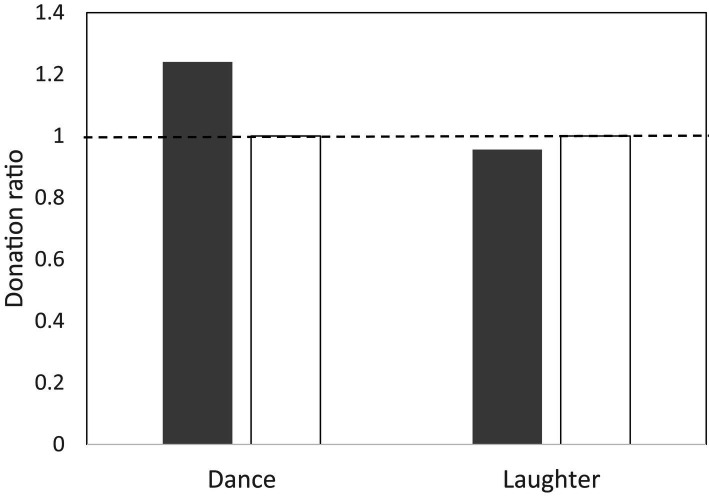
Mean ratio of monetary donation given to stranger versus that given to friend in a public good experiment following either dancing or laughter (watching comedy video; filled bars). Control groups (unfilled bars): unsynchronised gym sessions and watching non-comedy video. Group sizes are ~20 for dance groups and 4 (two pairs of friends) for laughter groups. Source: Tarr and Dunbar (2023) and Dunbar et al. (2021a,b).

**Figure 5:**
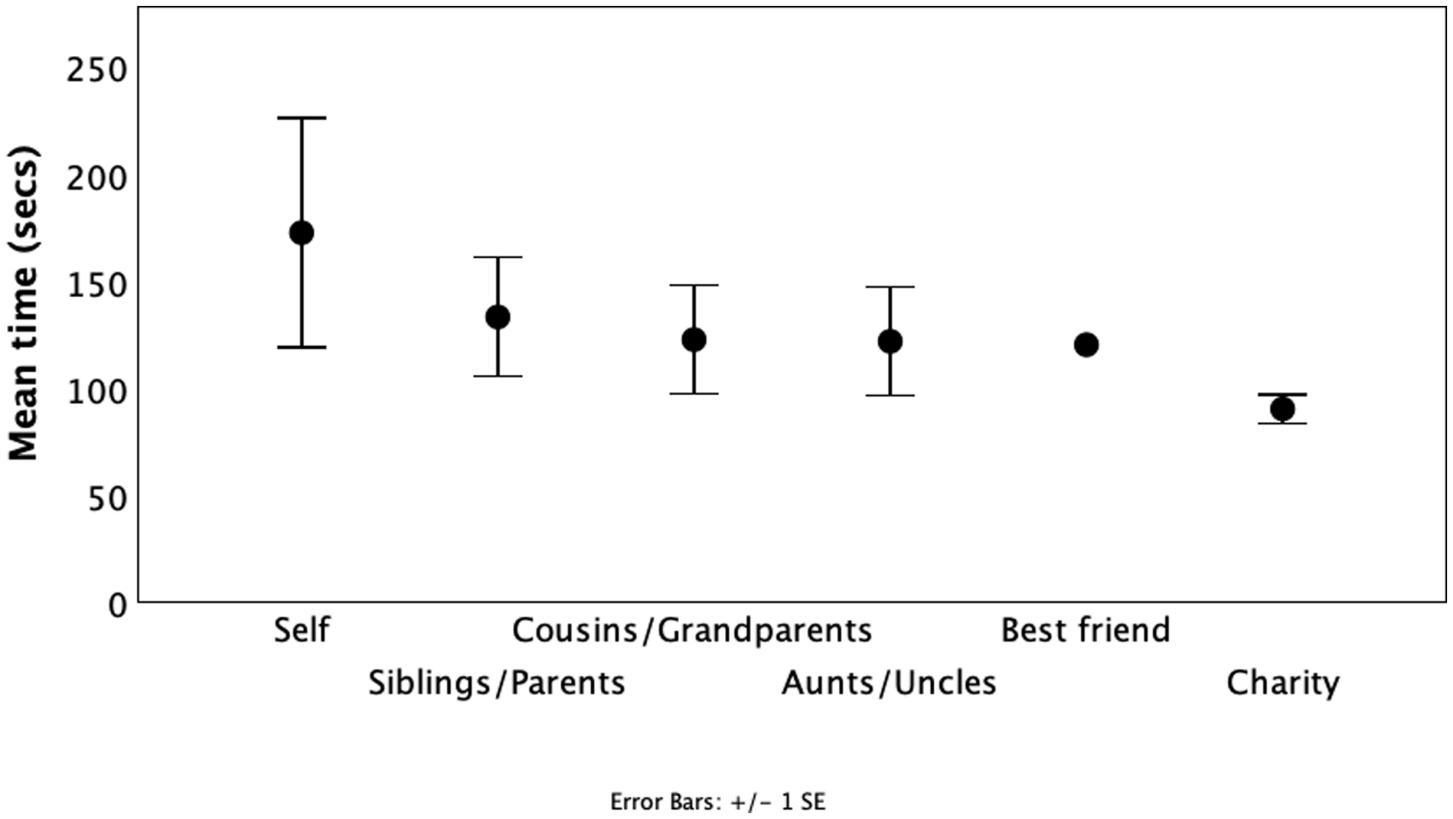
Mean (±1se) willingness to endure a painful exercise (seconds for which painful “Roman chair” ski training position was held) to benefit different individuals financially as a function of relatedness. Subjects performed the task six times for themselves as beneficiary, for three different named recipients from their family circle, for their named best friend or for a children’s charity. Source: Madsen et al. (2007).

This consistent tendency for bonding to increase after a musical activity contrasts strikingly with the pattern for altruistic behaviour. When participants in some of these experiments were invited to make a one-off donation to another member of their experimental group, they were no more likely to give generously after a musical session than after a non-musical control one (Figure 4). More importantly, this was also true even when the activity involved was another behaviour known to enhance bonding (e.g., laughter; Dunbar et al., 2021b). This suggests that prosociality (altruistic generosity to others) may be mediated by a completely separate neural system from social bonding. One reason for this lack of connection may be that altruism is a consequence, not the cause, of a bonded relationship. Most of our genuinely altruistic behaviour, including cooperation, is with people we already know well and hence have a bonded relationship with Figure 5. On the rare occasions that it occurs with strangers, it is with people whom we have reason to believe will behave honestly rather than cheat us – usually either because we can impose punishment on them if they do not or because a third party stands surety for their honesty. Cooperative exchanges with the individuals in the magic circle of our ~150 personal relationships (Dunbar, 2018, 2020) are given willingly without expectation of repayment. Beyond this magic circle, altruism and cooperation takes on a more explicitly transactional relationship that often depends on reputation (see also Hames, 1987; Berté, 1988; Panter-Brick, 1989; Dunbar et al., 1995). This may be the reason it has been so difficult to develop convincing models for the evolution of cooperation: all these models assume that we cooperate with (and behave altruistically towards) strangers, when in fact we rarely do so. We only act with genuine altruism towards strangers after they have been converted into friends through some bonding activity such as singing or dancing together.

## Why did music evolve?

So far, I have focussed on the role musical performance plays in social bonding. This does not necessarily mean that other functions may not play a role (or have not played a role in the past). Most human capacities are used in a number of different functional contexts: speech and language, for example, are used to convey useful information to others, to instruct children, to impress potential romantic partners as well as social superiors, to petition and beseech, and to compose fictional and other stories. Language cannot, however, have evolved under the selection pressure from all these different functions at the same time. A longstanding convention in evolutionary biology has been that one should be sceptical of any explanation that claims multiple selection processes because it is statistically unlikely that the selection effects will have been exactly equal for all these different functions. It is always better (until explicitly proven otherwise) to assume that one is the original selection pressure, and the others are either secondary selection effects (factors that come into play only once the trait has evolved because they yield minor fitness benefits that reinforce the main selection pressure) or windows of evolutionary opportunity (exaptations) or, alternatively, are constraints that evolution has had to find solutions for in order to allow the trait in question to evolve at all. A classic case of the latter concerns the correlation between brain size and diet in primates: though diet is frequently claimed to be the selection pressure for the evolution of large brains, in fact it is a solution to a constraint on brain growth (large brains are energetically expensive). Dietary shifts, and the cognitive skills that underpin more efficient diet choice, enable (but do *not* necessarily select for) the evolution of a large brain (Shultz and Dunbar, 2022). Unfortunately, far too many have fallen foul of this particular logical trap (Dunbar and Shultz, 2023a).

The capacity for musicking has been no exception. As I remarked at the outset, a plethora of possible functions have been offered for music-making: mate advertising (Darwin, 1871; Miller, 2000; Kaskatis, 2006), pleasure (Vuust and Kringelbach, 2010; Zatorre and Salimpoor, 2013), coordinating emotional states in groups of people (Roederer, 1984), coordinating (male) war bands (Hagen and Bryant, 2003), coalition signalling (Mehr et al., 2021) and, of course, social bonding (Dunbar, 2012; Dunbar et al., 2012; Savage et al., 2021). By its nature, mate choice is likely to have been a secondary function, most likely the outcome of sexual selection acting on an existing musical ability as a cue of gene quality. The induction of pleasure, or some other psychoactive state, is a mechanisms explanation, and intrinsically unlikely to be a functional end in itself. This is also true of synchronising emotional states, since the fitness benefit has to come from some emotional consequence of synchrony, not from the synchrony itself.

Coordinating war bands or signalling alliance size might be seen as plausible functions in their own right (Hagen and Bryant, 2003; Mehr et al., 2021), but they leave unanswered the question of why females ever bother to sing – and, more importantly, typically sing more and better than males do in everyday contexts (Welch et al., 2012). Music certainly features in the context of some forms of combat (football matches, pre-modern forms of battle where opposing arms line up against each other), but these contexts are very late in evolutionary terms (within the last few thousand years at most). In small scale hunter-gatherer societies (the context in which music inevitably evolved), almost all conflicts take the form of raids, and raids are invariably conducted in absolute silence because the success of a raid depends entirely on surprise.

The Maori *haka* provides an instructive example. Although in fact associated with many different social contexts (including welcoming dignitaries, funerals and other major social events), one version of it (the *peruperu*) is essentially a war dance intended to intimidate the opposing side. However, watching this version of the *haka* being performed (e.g., by the New Zealand All Blacks national rugby team at the start of every international match) makes it obvious that the function is much less about intimidating the opposition and much more about lifting the performance of the home side. The endorphin surge produced by the combination of call-and-response unison singing and the highly synchronised, highly tensed, stomping dance-like movements must inevitably lift pain thresholds dramatically (as in Figure 1), and create a sense of brotherhood (as in Figure 3) – a feature that is, by the way, inculcated into the All Blacks mindset from the moment a player joins the squad (Eastwood, 2021). In real war, a war dance may well significantly reduce the risk of individuals deserting when the going gets tough (the main problem that small scale armies face). That said, even if the *haka* does intimidate the opposition, the Maoris, like all horticulture-based societies, are an historically late phenomenon, where warfare took the form of drawn-up battle lines. If it intimidates the opposition in doing so, that’s an added bonus (a secondary selection pressure).

That singing may have pain-relieving benefits is well illustrated by the very long history of sea shanties (used mainly when hauling up heavy sails and anchors on sailing ships, or when rowing in medieval galleys), and by a wide variety of other forms of work song including Scots Gaelic waulking songs (call-and-response songs used by groups of women to reduce the physically tiring tedium of stretching hand-made tweed cloth), field songs (traditional in West Africa and adapted by American plantation slaves) and perhaps even hunting songs, almost all of which have the same call-and-response structure (Hugill, 1961; Johnston, 1973; White and White, 2005; Gioia, 2006).

The bonding role of singing is implicit in its likely origins either in motherese (the distinctive song-like communication between mothers, in particular, and very young infants and in nursery rhymes in all cultures: Dissanayake, 2001; Falk, 2004; Malloch and Trevarthen, 2018) or in primate contact calls (Mehr et al., 2021). Though New World callitrichids are a melodic exception, most primate contact calls are quiet grunts [and *not* loud barks or “lost” calls, contra Mehr et al., 2021] exchanged between grooming partners so as to allow allies to maintain close spatial coordination when foraging through dense vegetation. On its own, contact calling lacks most of the key criteria for music (variation in pitch, communal performance). However, in gelada, contact calling can sometimes build into a distinctive form of chorusing to which all the females in the harem contribute (with the male adding a coda). Gelada contact calls are also unusually variable (compared to those of other catarrhine monkeys and apes), and under certain circumstances can express an emotional element of excitement. It may be no coincidence that gelada live in unusually large groups by primate standards. These choruses are most similar to the group howls of howler monkeys: usually interpreted as territorial calls, these are unusual by the standards of primate territorial calls (which are normally only given by one adult male) in that the entire group takes part, building to an incredible crescendo of noise (Sekulic, 1982). In gelada, these chorusing events do seem to substitute for grooming while animals are preoccupied with foraging in contexts where they can all too easily become separated from each other in herds of several hundred animals. In this respect, these choruses are functionally very similar to the chorusing of those species of birds that live in stable, bonded groups (Guinea fowl, starlings, mousebirds, babblers, parrots) and the (decidedly more musical) antiphonal duets of pairbonded tropical boubous and other bushshrikes when foraging separately in dense scrubland (Thorpe, 1972; Hall, 2004, 2009; Wheeldon et al., 2020, 2021).

The role of music in social bonding surfaces in another interesting context, namely the curious phenomenon of octave equivalence (Bannan et al., 2023). Octave equivalence refers to the fact that men’s and women’s natural singing voices are exactly an octave apart. While women and all children sing in the same register, men’s voices are in a much lower register. This is usually attributed to sexual selection for larger body mass in men and is assumed to reflect the role that body size plays in male–male competition over reproductive access to females (Puts et al., 2006, 2016; Aung et al. 2023). While it is surely the case that the males’ lower voices reflect their larger body size (indeed, stature is a strong criterion underpinning women’s mate choice decisions: Pawlowski et al., 2000), this alone cannot explain the magnitude of the quantitative difference in fundamental frequency between the sexes. Human males have *much* deeper voices than we would expect for their body size (Bannan et al., 2023: Figure 1). Were human males to have a body size appropriate for their voice register, they would need to be ~3 m tall. In other words, while sexual dimorphism in body size might well have kick-started the dimorphism in voice register, something else seems to have greatly exaggerated the effect.

Bannan et al. (2023) argue that the key lies in octave equivalence: this has the effect of allowing men and women to sing in harmony. In effect, it permits singing in a form of unison, thereby enhancing the “tingle factor” of singing in much the same way that plainsong (Gregorian chant, a well known form of unison singing) does. It is difficult to see why this might be selected for in the context of pairbonding, since couples do not often sing together in isolation. But they do sing together as part of a group, and do so frequently. Although dancing competence and synchrony certainly plays a role in mate choice (see, for example, Pitcairn and Schleidt, 1976), this is open to the obvious interpretation that synchronised dancing elevates endorphin levels and (demonstrably) makes us more willing to acquiesce in a mating invitation. In reality, we much prefer to sing in groups, making it much more likely that this is the context in which both singing first evolved and its technical perfections were finessed in order to enhance group cohesion.

## When did musicality evolve?

**Figure 6:**
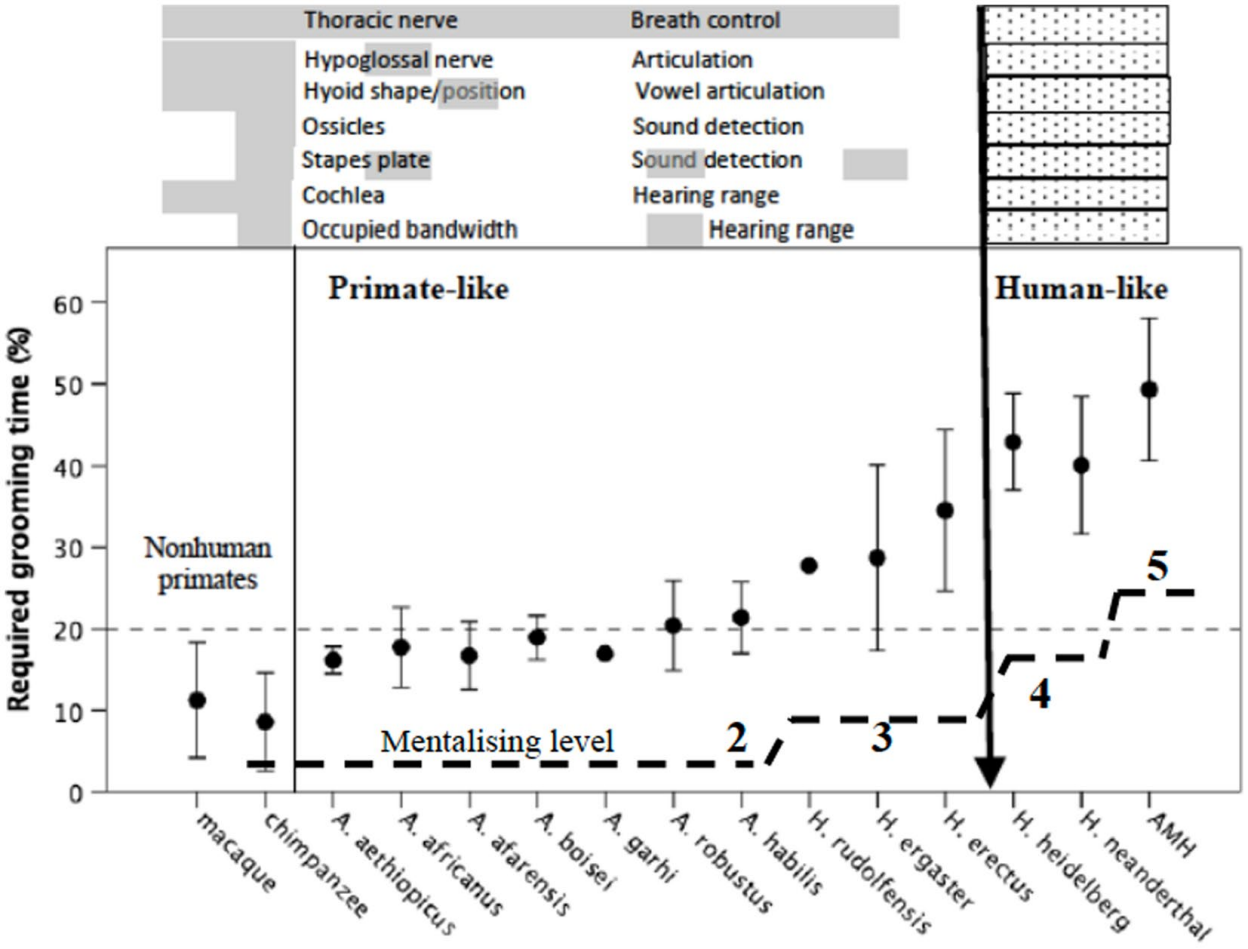
Mean (±95% CI) estimated grooming time requirement for the main hominin species, and two representative Old World primates calculated in the same way. Grooming time is estimated from group size, using the equation given by Dunbar (1991); group size is estimated from neocortex ratio using the hominid grade regression line from Dunbar and Shultz (2021b), with this in turn estimated from cranial volumes using the equations given by Aiello and Dunbar (1993). Estimates are calculated separately for individual fossil crania. Short dashed line indicates maximum observed value for grooming in modern primates (and observed value of social interaction for contemporary humans), all determined from observed activity budgets. Estimated group size are given in Gowlett et al. (2012). Above the graph are listed seven anatomical indices of speaking and hearing that differ between modern humans (hatched bars) and all other primates (grey bars) whose presence/absence can be documented in at least some fossil hominins (source: Dunbar, 2009; Bannan et al., 2023). Long dashed lines indicate estimated mentalising competences (indexed as mean maximum achievable level of intentionality) for the same species; estimates are based on an equation relating known mentalising levels to prefrontal cortex volume in living humans, apes and monkeys (source: Dunbar, 2014).

Given that grooming is the principal means that anthropoid primates use to create the glue that holds groups together (Dunbar, 2023), we need to determine the likely grooming time requirement that individual populations faced over the evolutionary history of the hominins. If we can do this, we can then undertake a reverse engineering analysis and determine when they would have run out of available grooming time and, hence, had to introduce a novel bonding strategy. Since we know that grooming time requirement is a simple linear function of social group size (Dunbar, 1991; Lehmann et al., 2007; Dunbar et al., 2013), and group size is a very straight forward linear function of neocortex size (Dunbar, 1998; Shultz and Dunbar, 2022; Dunbar and Shultz, 2023a,b), this is simply a matter of interpolating through the relevant equations (see Dunbar, 2014, 2022a). Group size estimates for individual populations of the major hominin species (based on endocranial volumes for individual fossil specimens) are given in Gowlett et al. (2012) and Dunbar (2022a). The corresponding estimates for grooming time requirements are given in Figure 6 as mean ± 95% CI for individual species. We seek the point in human evolution where the demand for the conventional bonding mechanism (social grooming) significantly exceeded the animals’ capacity to meet this demand, since this will identify the point where a new adaptation is needed.

The distribution in Figure 6 suggests three major pinch-points at which novel “grooming” behaviours might have appeared. One is at the transition from australopithecines to early *Homo* around 2 Ma (where mean group size increased from around 50 to 75); the second is the transition from early *Homo* to archaic humans sometime around 500 ka (where mean group size increased from about 75 to about 120); and the third is the transition from archaic humans to anatomically modern humans (*Homo sapiens*) around 250 ka (where mean group size increased from ~120 to 150).

It is important in this context to appreciate that australopithecine time budgets were at their limit, with no spare capacity (Bettridge, 2010; Bettridge and Dunbar, 2023, submitted). Note, by the way, that rest time does *not* represent spare capacity that can be used to subsidise other activities: it is downtime forced on animals by the thermal environment (Korstjens et al., 2010; Dávid-Barrett and Dunbar, 2016), and cannot be used even for grooming due to the fact that grooming generates heat (Yetish et al., 2015; Monsivais et al., 2017). This means that an increased demand for social grooming is only possible if adjustments can be made to other components of the time budget (principally feeding and travel; Dunbar et al., 2009) or by increasing the number of individuals who can be groomed simultaneously (the “broadcast group” size) so as to make more efficient use of what time is available (Dunbar, 2014).

While some time savings may have been possible through a shift in diet (more meat) and a longer stride (less travel time required), these would only have made very modest contributions (Dunbar, 2014). The principal source of time savings is more likely to have been social time, and this would have depended on being able to increase the number of individuals who could be groomed simultaneously. The intimacy of physical grooming makes it a strictly one-on-one phenomenon, even in humans, so increasing the number of individuals that could be simultaneously groomed is not a feasible option (Dunbar, 2022a). Instead, humans seem to have found a number of ways to trigger the endorphin system so as to be able to “groom” virtually with several individuals at the same time. These include laughter, singing, dancing, feasting, storytelling, and the rituals of religion – a suite of behaviours that constitute a human social toolkit. All of them have been shown both to up-regulate the endorphin system and to elevate the sense of social bonding (Dunbar, 2021a). Their interaction groups vary in size between three (laughter: Dezecache and Dunbar, 2012) and several hundred (singing: Weinstein et al., 2016; religion: Dunbar and Sosis, 2018; Dunbar, 2021b), and are thus considerably larger than those for grooming in both primates and humans (where the broadcast group size is one; Dunbar, 2022a).

I have argued that laughter is the most likely solution to the first transition (Dunbar, 2022a). It is the only one of the six behaviours that has a strong involuntary component (suggesting a strongly hardwired basis and a deep history), is shared with the great apes (albeit highly modified), is highly contagious and does not need a stimulus, has the most limited broadcast circle (the number of individuals who can be reached at any given moment: three), does not require language, and seems to function in a very similar way to other forms of chorusing in primates (e.g., roaring in howler monkeys, group contact calling in gelada) and the “flock talk” of group-living birds (Riters et al., 2019). It is important to note that laughter and singing operate on very different anatomical principles. Laughter (and coughing) involves heavy, pulsed exhalations involving strong contractions of the chest wall and diaphragm muscles under the control of the phrenic (C_4_) and vagus (C_10_) nerves (Collis et al., 1954; Widdicombe, 1995). In contrast, singing (and speech) requires long, slow, controlled exhalations, and is under the control of the thoracic T_3-5_ nerves (Wild et al., 2003; Mac Larnon and Hewitt, 2004). In effect, laughter has all the right characteristics to be a form of wordless group chorusing.

Conversely, feasting, storytelling and the rituals of religion all depend on complex language (of the kind we find in anatomically modern humans). Singing (*qua* wordless chorusing) and dancing seem to form a separate group since they are not dependent on language (though singing does depend on the same breath control as language) and, like laughter, have a certain level of contagiousness and spontaneous emotionality.

We might be able to pinpoint the origins of these two later groups of bonding activities if we can specify when speech and fully modern language first appeared. This might seem a challenge, but in fact we are in a rather better position to do this than might appear at first sight. There are a number of anatomical markers for speech that relate to control over breathing, articulation and hearing range. These markers are all much larger relative to body size (or are positioned differently) in modern humans compared to all other primates, and the corresponding values for a number of fossil hominins have now been determined. These are plotted on the upper part of Figure 6 for both hominins and two representative Old World primates.

It is important to appreciate that this only tells us about the ability to articulate and hear human-like sounds, not whether or not the species concerned had language. The capacity to formulate and interpret fully modern language depends on high level mentalising skills (the ability to understand the mind states of one’s listeners). In normal human adults, semantic competence correlates with mentalising competence (Dunbar, 2014; Oesch and Dunbar, 2017), not least because they share the same neural network (Hervé et al., 2013). In modern humans, normal adult function corresponds to fifth order mentalising (in effect, the capacity to handle four other people’s mental states as well as one’s own: Stiller and Dunbar, 2007). However, mentalising competences vary among normal adults between about third order and sixth order (Kinderman et al., 1998), and this directly affects the ability to enjoy fictional stories (Dunbar, 2005; Zunshine, 2006, 2022; Carney et al., 2014), the ability to appreciate jokes (Dunbar et al., 2016a,b; Dunbar and Stirling-Middleton, 2023) and the understanding of religious propositions (Dunbar, 2022b). Mentalising competences are in turn directly correlated with the volume of core prefrontal cortex regions that manage mentalising skills both within humans (Powell et al., 2010, 2012) and across primate species (Dunbar, 2009; Dunbar and Shultz, 2021b). In both cases, mentalising ability correlates with brain prefrontal lobe size, and we can use this relationship to estimate the mentalising competences of fossil hominins. These are plotted as the dashed line on the lower part of Figure 6.

Figure 6 suggests that the anatomical markers for speech all change from primate-like to human-like at around the same time (the appearance of archaic humans, *Homo heidelbergensis*). This suggests that the capacity for human-like speech cannot have appeared earlier than this. This means that archaic humans (Heidelbergs and Neanderthals) could well have had language. However, it could not have been fully modern language: fully modern mentalising capacities (fifth order mentalising) were only achieved with anatomically modern humans (*Homo sapiens*). Archaic humans are likely to have had a mentalising capacity similar to that of human teenagers (fourth order mentalising). While this is more than sufficient to allow a competent conversation, it would have radically limited the quality of their storytelling, their jokes and the philosophical complexity of their ideas. Note, by the way, that this does not tell us anything about their intelligence in the sense of IQ, which may well have been within the modern human range. Rather, the issue is *social* intelligence.

One obvious interpretation of the data in Figure 6 is that the seven speech markers identify the appearance of singing. Singing and dancing have a major advantage over laughter in respect of their much larger broadcast group sizes. Weinstein et al. (2016), for example, found that endorphin activation and bonding were significantly higher in choirs of 200 than in choirs of 20. Robertson et al. (2017) reported that naturally forming freestyle dance groups averaged 4.4 individuals at any given moment, with a cumulative group size (all the individuals that engaged with each other during the course of one dance) of 7.1; in contrast, conversation groups averaged 3.4, with a cumulative mean group size over time of just 3.7. One reason is that, in the absence of the need to hear what a speaker is actually saying, more individuals can coordinate their actions, no matter how noisy the environment. These differences between laughter and musical activity, on the one hand, and the language-based ones, on the other hand, make singing and dancing by far the more likely candidate to fill the bonding gap for the second transition at around 500 ka, predating the appearance of fully modern language by at least 200,000 years.

## Discussion

I summarised a series of experiments that tested the hypothesis that musical performance (singing, dancing and playing instruments) plays a seminal role in bonding individuals into groups. These experiments show that musicking activates the brain’s endorphin system (the mechanism that underpins grooming-based bonding in primates) and through this creates an elevated sense of bonding, or belonging (entiativity). This sense of bonding seems to be specific to those with whom one engages in the activity, and does not affect relationships even with close friends if these are not physically present. Furthermore, it seems that this process has nothing directly to do with cooperation or prosociality. Rather, cooperation arises later as an indirect consequence of the bonded relationships in what is a two-step dual mechanism: creating bonded groups provides a platform within which cooperation occurs as a matter of course (in effect, through personal commitment). The bonded relationship has to be established well before any need for cooperation: upregulating the endorphin system does not, of itself, lead to immediate prosociality. That requires repeated exposure to establish a personalised relationship, and that process is very time-expensive. In the absence of close bonding, cooperation and altruistic behaviour become more explicitly transactional and less spontaneous.

Most studies of the evolutionary aspects of behaviour in humans overlook the fact that living in groups is a crucial part of the primate (and human) evolutionary strategy, and hence ignore its importance as the motor of primate evolutionary success. Instead, most analyses focus on short-term objectives, emphasising individual benefits. At the same time, they invariably fail to appreciate that group-living is fraught with difficulty and has been a major challenge for primates (including humans) throughout their history. Taking a wider, more biological perspective allows us to see how solving the problem of group-living allows animals to manage environmental threats at the group level in ways that greatly reduce the cost to the individual (Dunbar and Shultz, 2023a,b). At the same time, it reminds us that group-living comes with costs and that much of what animals do is designed to minimise these costs (Dunbar and Shultz, 2021a).

Perhaps because of this misdirected focus, studies of the function of music-making have struggled to find convincing individual-level reasons why it might have evolved, with some, notoriously, feeling obliged assert that it has no function at all (Pinker’s “auditory cheesecake” claim). An analogous problem has bedevilled studies of the evolution of cooperation. Most have struggled to find convincing explanations as to why cooperation might have evolved either because they have viewed it as a simple immediate-return transaction or because they have viewed it as the cause of group-living. Neither claim is true. Group-living is a coordination problem, not a cooperation problem. Hence the importance of close-quarter contact calls as a form of “grooming-at-a-distance” in anthropoid primates (Arlet et al., 2015), as well as other mammals (elephants: Soltis et al., 2005; meerkats: Engesser and Manser, 2022) and birds (Perez et al., 2015; Fernandez et al., 2017) that live in bonded groups. In fact, cooperation is a beneficial by-product (or consequence) of living in groups, not its cause: once you have bonded groups, the public goods dilemma costs that bedevil cooperation cease to exist (Dávid-Barrett and Dunbar, 2013). Seen as a solution to a group-coordination problem, music-making makes perfect evolutionary sense. It also makes sense of the endorphin and bonding effects that we observe – something that otherwise appears to be a case of genuinely non-functional auditory cheesecake.

Like laughter, music-making (in all its forms) is a form of virtual social grooming designed to supplement conventional grooming so as to allow larger groups to be bonded (Dunbar, 2022a). The evidence that musicking in synchrony dramatically enhances the endorphin effect, and that the same endorphin up-regulation is observed in birds when they engage in group vocalising (Riters et al., 2019), reinforces this conclusion. That music evolved before language is suggested by its visceral nature: like laughter, we respond to music subconsciously in ways that do not depend on language and which are often difficult to express in words. This does not mean that language did not subsequently intrude into music. Just as we found ways to exploit language in the form of jokes so as to trigger laughter, so we added words to the music to create songs or offer programme notes that help an audience interpret orchestral music. In this respect, music exploits the storytelling mode of the “Seven Pillars of Friendship” (Dunbar, 2018) in ways that enhance the cultural meaning of the music (whether this be national anthems or romantic ballads).

Singing (even without words) and laughter are not, however, the same. They depend on very different anatomical mechanisms. Singing represents an entirely novel evolutionary development that emerged a million and a half years after laughter. Importantly, however, the breath control that underpins singing played a central role in the evolution of language some 300,000 or so years later: both depend on the ability to control long slow exhalations. The musicality of singing (as in motherese) may well have been instrumental in creating the rhythmic and rhyming properties of language, with the natural breathing patterns associated with singing influencing the segmented structure of multi-clause sentences.

## Data availability statement

Publicly available datasets were analyzed in this study. All the data used in this manuscript are available in the published papers cited in the text.

## Ethics statement

The studies involving humans were approved by University of Oxford Combined University Research Ethics Committee. The studies were conducted in accordance with the local legislation and institutional requirements. The participants provided their written informed consent to participate in this study.

## Author contributions

RD: Conceptualization, Formal analysis, Funding acquisition, Investigation, Methodology, Project administration, Supervision, Validation, Writing – original draft, Writing – review & editing.
